# Pli falciforme rétinien

**DOI:** 10.11604/pamj.2014.17.122.3944

**Published:** 2014-02-21

**Authors:** Hakima Elouarradi, Rajae Daoudi

**Affiliations:** 1 Université Mohammed V Souissi, Service d'Ophtalmologie A de l'hôpital des Spécialités, Centre Hospitalier Universitaire, Rabat, Maroc

**Keywords:** Pli falciforme rétinien, œil, décollement de rétine, retinal fold, eye, detached retina

## Image en medicine

Les plis falciformes rétiniens font partie des dysplasies vitréorétiniennes. Ils se présentent comme un pli tendu de la papille vers une masse blanchâtre située à l'extrême périphérie rétinienne. Nous rapportons l'observation d'une patiente âgée de 24 ans, qui présente un pli falciforme rétinien au niveau de l'oeil droit. Il s'agit en fait d'une atteinte ophtalmologique rare de pathogénie controversée. Peuvent être présents dans les rétinopathies des prématurés, la maladie de Norrie, l'incontinentia pigmenti, le vitréorétinopathie dominante. Ils ont été décrit dans certaines pathologies liées à l'X et dans d'autres syndromes malformatifs. Les complications les plus fréquentes sont le décollement de rétine tractionnel et la néovascularisation rétinienne. Le traitement des plis falciformes est fonction de l'affection causale.

**Figure 1:**
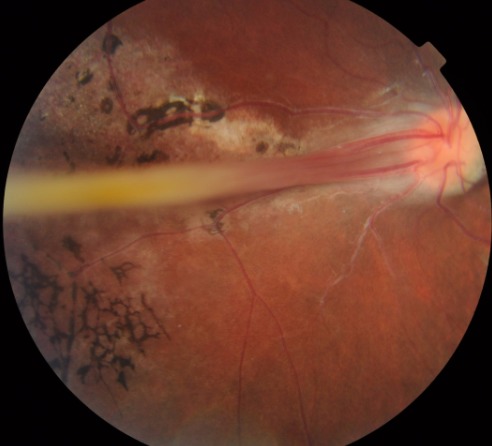
Pli falciforme rétinien

